# A giant solitary fibrous tumor of the abdominal pelvic cavity: A case report and literature review

**DOI:** 10.1097/MD.0000000000039270

**Published:** 2024-08-09

**Authors:** Cao Li, Jianyang Yang, Hongli Chen, Lie Yang

**Affiliations:** a Division of Gastrointestinal Surgery, Department of General Surgery, West China Hospital, Sichuan University/West China School of Nursing, Sichuan University, Chengdu 610041, China; b Department of Gastrointestinal Surgery, Division of Gastroenterology, West China Xiamen Hospital of Sichuan University, Xiamen, China; c Department of Intensive Care Unit, West China Hospital of Sichuan University, Chengdu, China; d Institute of Digestive Surgery, State Key Laboratory of Biotherapy and Cancer Center, West China Hospital of Sichuan University, Chengdu, Sichuan, China.

**Keywords:** abdominal pelvic cavity tumor, case report, solitary fibrous tumor, surgical resection

## Abstract

**Rationale:**

Solitary fibrous tumor (SFT) is a rare mesenchymal tumor, especially the giant one from the abdominal pelvic cavity. We report on a rare case of a giant SFT of the abdominal pelvic cavity to review the existing literature in detail to improve the diagnosis and treatment of SFT.

**Patient concerns:**

The patient is a 52-year-old female who presented with 2 weeks of abdominal distension. Abdominal magnetic resonance imaging showed a giant mass (>20 cm) in the abdominal pelvic cavity, considered a mesenchymal tumor. She denies a history of tumor disease.

**Diagnoses:**

A whole abdomen bulge and a mass of about 18 cm × 10 cm on the right side and middle side were found in the physical examination after admission. Abdominal enhanced computed tomography revealed a giant cystic-solid mass located on the middle and right side of the abdominal pelvic cavity, measuring approximately 20.4 cm × 11.7 cm, with multiple cystic changes and necrosis and compression of adjacent organs and tissues, and marked inhomogeneous enhancement.

**Interventions:**

The patient underwent an open abdominal pelvic cavity giant tumor operation to achieve a radical resection, and did not undergo chemotherapy or radiotherapy.

**Outcomes:**

The patient underwent open complete resection of a giant abdominal pelvic tumor with no complications and was diagnosed as SFT according to the pathology, immunohistochemistry showed that the tumor tested positive for CD34(+), STAT-6(+), and Ki-67 (10%). Abdominal computed tomography scans were performed 6 months after resection, and no signs of recurrence or metastasis were found.

**Lessons:**

The clinical symptoms and imaging features of giant abdominal pelvic cavity SFT are not typical. Preoperative diagnosis is difficult and has the potential for malignancy. Based on the results of the current study, there is no standard treatment strategy around the world and the therapeutic effect of radiation therapy and chemotherapy is relatively limited. Thus, complete surgical resection and close clinical follow-up are advocated.

## 1. Introducltion

Solitary fibrous tumor (SFT) is a rare mesenchymal tumor originated from CD34 positive dendritic mesenchymal cells that may occur virtually anywhere throughout the body,^[1,2]^ within the abdominal pelvic cavity are particularly rare, especially giant tumors (up to 20 cm in diameter).^[3–5]^ The imaging features of SFT are many and varied, and preoperative diagnosis is difficult. There is no consensus on treatment methods, and no definite data on recurrence and metastasis of SFT.^[6]^ Therefore, we report on a rare case of a giant solitary fibrous tumor of the abdominal pelvic cavity to review the existing literature in detail to improve the diagnosis and treatment of SFT.

## 2. Case report

### 2.1. Patient information

A 52-year-old female patient was admitted to the hospital with 2 weeks of abdominal distension. The patient did no display any other symptoms such as chills, fever, cough, nausea, vomiting, abdominal pain, diarrhea, anorexia, fatigue, and bleeding stool. She had no history of malignancy or surgery.

### 2.2. Clinical findings

The physical examination showed the whole abdomen bulge, soft, the right upper abdomen and middle side could touch a mass of about 18 cm × 10 cm, its texture was pliable, and the activity was moderate.

### 2.3. Diagnostic assessment

**Figure 1.**
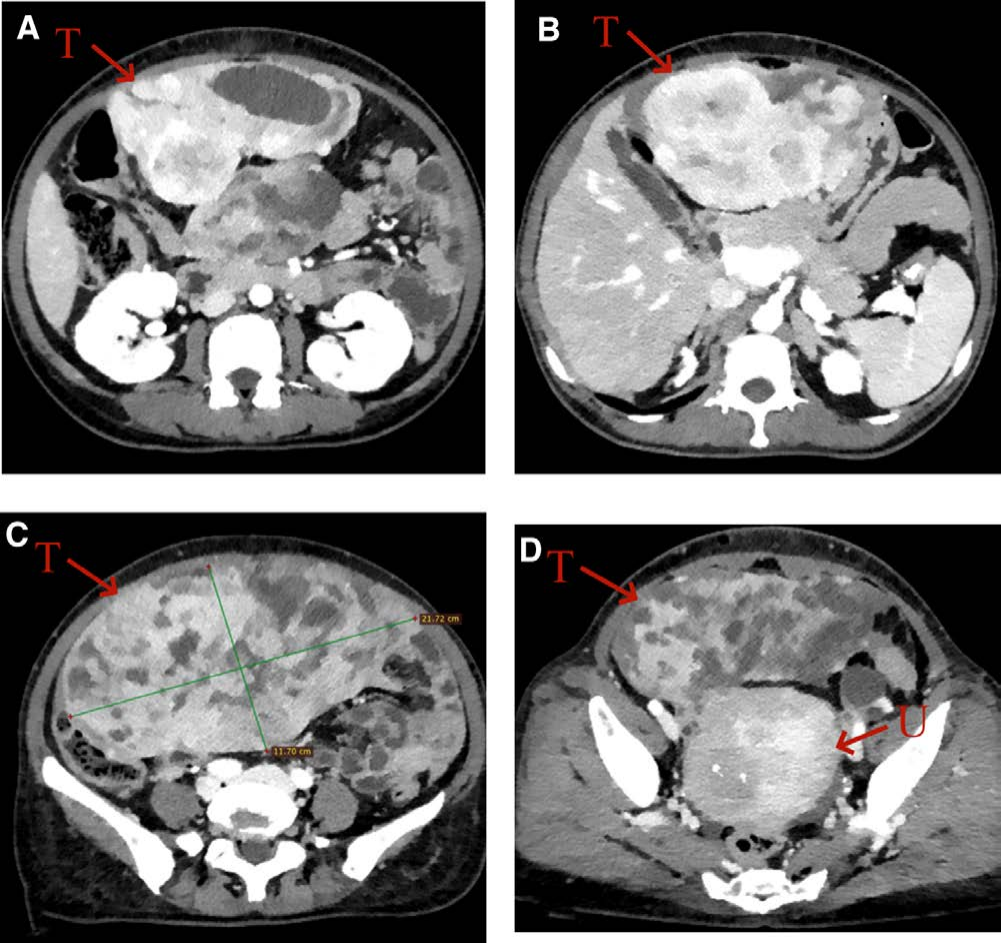
Enhanced CT of the abdomen. (A) and (C) Preoperative enhanced CT: the axial plane of the abdominal pelvic tumor. (B) Postoperative CT: no evidence of recurrence and metastasis. T = tumor, U = uterus.

Except for the significant increase in result of CA125 (124 U/mL), other laboratory test including complete blood counting, biochemistry, coagulation function, and other tumor markers such as CEA, CA199, and CA724 were within normal limits. Abdominal enhanced computed tomography (CT) demonstrated a giant cystic-solid mass located on the middle and right side of the abdominal pelvic cavity, measuring approximately 20.4 cm × 11.7 cm, with multiple cystic changes and necrosis and compression of adjacent organs and tissues, and marked inhomogeneous enhancement. Considering the possibility of mesenchymal tumor (Fig. 1A–C). Regional lymphadenopathy and distant metastasis (liver, brain, lung, bone, and bone marrow) were not noted. Preoperatively, we considered this tumor can be resection by surgery.

### 2.4. Therapeutic intervention, follow-up, and outcomes

**Figure 2.**
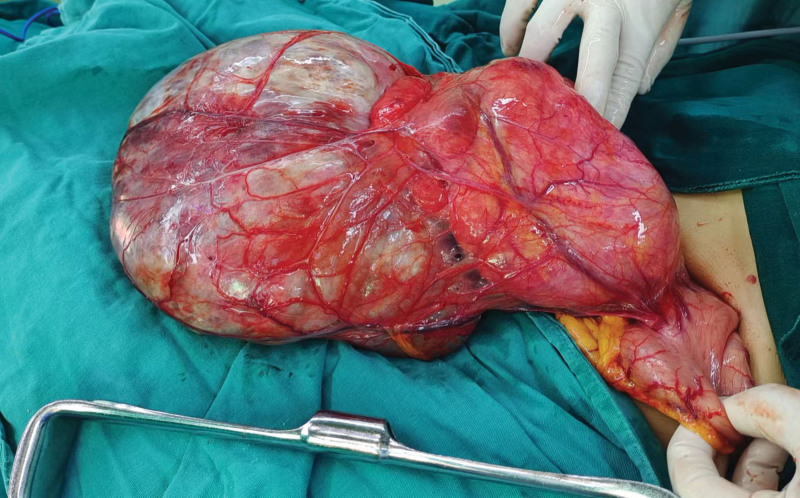
The intraoperative appearance of the tumor.

**Figure 3.**
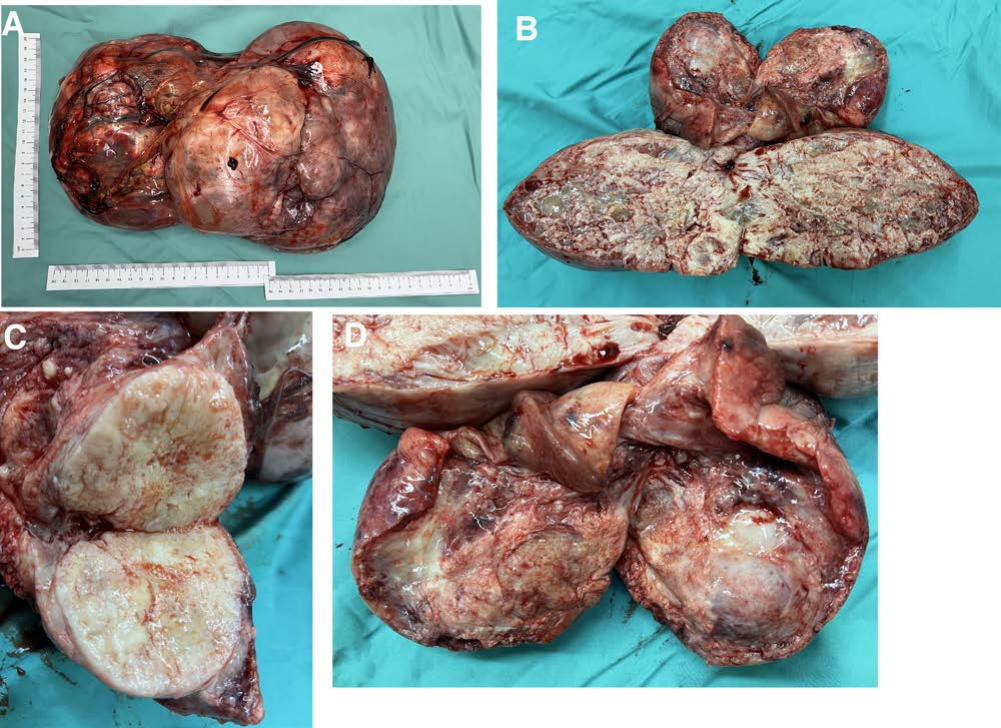
The macroscopic views of the tumor. (A)–(D) The tumor was firm, well-capsulated and omental tissue was visible, with a grayish-white cut surface, tremelloid, with visible bleeding, and had localized cystic cavity-like changes.

**Figure 4.**
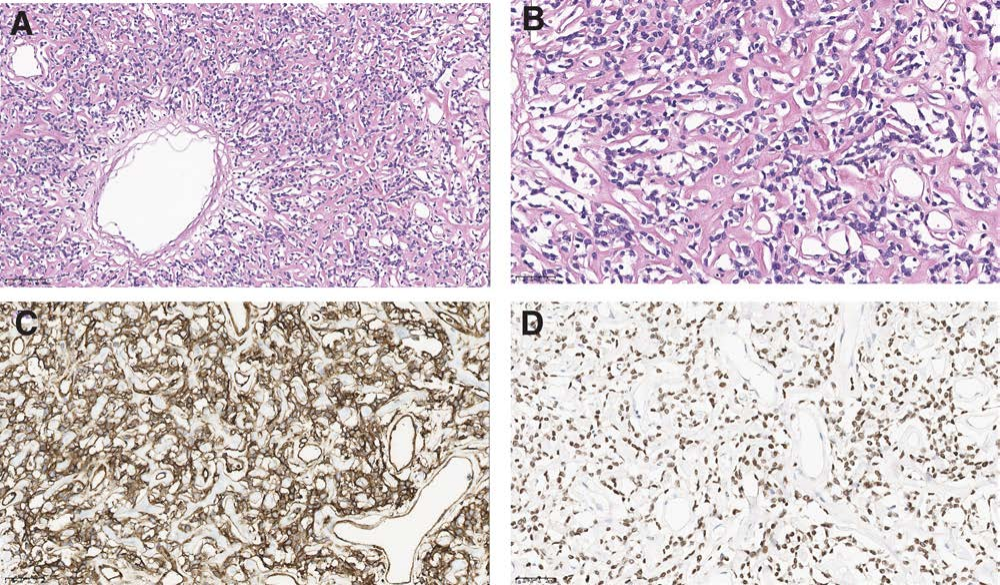
The microscopic views of the tumor and its immunohistochemical examination. (A) At low magnification, the tumor consists of sparse areas of spindle-shaped tumor cells, varying amounts of vitreous collagenized stroma and unique vascularization (staghorn-like structure) (HE 200). (B) At high magnification, the tumor cells were spindle-shaped, with homogeneous nuclear chromatin and positive staining for nuclear markers (HE 400). (C) CD34-positive and diffuse expression in tumor cells (SP 400). (D) STAT-6 showed positive and diffuse expression in tumor cells (SP 400).

The patient had a complete preoperative examination, without contraindications to the operation (Fig. 2). Open complete resection of giant abdominal pelvic tumor was performed with no complications. Abdominal CT scans were performed at 6 months after resection, and no signs of recurrence or metastasis were found (Fig. 1D). A macroscopic examination revealed a 33 cm × 21 cm × 11 cm tumor (Fig. 3A–D) that was firm, well-capsulated, and omental tissue was visible, the cut surface was grayish-white, tremelloid, with visible bleeding. Histopathology showed margins were negative, The tumor is characterized by spindle cells with varying amounts of hyalinized collagen, alternating sparse and abundant cellular areas, and a branching hemangioepithelioma-like vascular pattern (Fig. 4A). The high magnification showed that the tumor contained spindle cells, with less cytoplasm appeared red and uniform nuclear staining, the tumor cells were surrounded by abundant collagen fibers, without cellular atypia and mitotic figures (Fig. 4B). Immunohistochemistry showed that tumor tested positive for CD34(+) (Fig. 4C), STAT-6(+) (Fig. 4D), and Ki-67 (10%) and negitive for SMA, Desmin, S-100, EMA, DOG1, CD117, TLB1, CDK4, Pax-8, MDM2, Myogenin, and CD31. Combined with histopathology and immunohistochemical results, SFT (medium risk) was diagnosed.

## 3. Discussion

SFT originates from CD34-positive dendritic mesenchymal cells and is capable of differentiating into fibroblasts, myofibroblasts, and vascular epithelial cells.^[6]^ It is found in almost any part of the body, often involves the thoracic cavity (pleura, lung), also occur in other locations such as the abdominal pelvic, mass, retroperitoneal, and pelvic.^[7]^ Although abdominal pelvic SFT is not uncommon, those giant SFT are very rare. As abdominal pelvic SFT are usually asymptomatic until they reach a large enough size to cause a compressing effect on other organs.^[8]^ In this case, the patient had no obvious clinical symptoms.

Ultrasound of the SFT in the abdominal pelvic typically shows an echogenic mass with low or heterogeneous echogenicity with well-defined borders.^[9]^ In this case, ultrasound showed a mass with ambiguous borders with irregular morphology, heterogeneous echogenicity, the lesions are nodular and fused, and visible small anechoic areas, considering the tumor with cystic, hemorrhagic or necrotic areas. CT scan usually showed a heterogeneous enhancement of nodular or mass during the arterial phase, along with cystic, hemorrhagic, or necrotic areas.^[10]^ The enhancement of the mass gradually increase in the venous and delayed phases and the density of the lesion became more homogeneous with a “fast-in, slow-out” pattern.^[9]^ The CT findings in this case were consistent with the literature. The abdominal pelvic SFT usually showed solitary lesions with clear borders on magnetic resonance imaging, with low signal intensity on T1 WI and mixed signal intensity on T2 WI.^[11]^ This case showed a gourd shaped mass with isointensity or slightly low signal intensity on T1 WI and high signal intensity on T2 WI, and the part of parenchymal components became visible after lesion enhanced.

The gross specimen of abdominal pelvic SFT is usually a isolated solid mass, well-defined, complete capsule, elliptic, gourd shaped, or irregular ambulated shape.^[12]^ The cut surface of SFT was gray, yellowish gray, even tan in color, fish-flesh, with visible necrotic, hemorrhages, gelatinous, and cystic changes.^[13]^ Histology revealed that the SFT usually consists of spindle cells with mild to moderate nuclear polymorphism with mitotic rates ranged from 0/10 to 30/10 HPF, and mitotic was rare.^[14]^ The traditional SFT was typical of hypervascular vessels with thin-walled vessels with staghorn vessels.^[10]^ SFT was characterized by the presence of a mucus-like interstitial matrix with alternating sparse and abundant cellular areas, coarse collagen fibers, and a branching hemangioepithelioma-like vascular pattern.^[15]^ Numerous studies^[16–19]^ have shown that nuclear atypia, high mitotic count (>4/10 HPF), tumor size >10 cm, tumor sites, positive margins and necrosis, or hemorrhagic are the most reliable indicators of poor prognosis. The latest immunohistochemical study showed that CD34, CD99, Bcl-2, and STAT-6 were previously thought to be the most useful positive immunohistochemical markers for SFT, with a high degree of specificity and sensitivity.^[20]^ According to morphology, histological, and immunohistochemistry results, it is clear that this case meet the diagnostic criteria for the classical medium-risk SFT.

The primary treatment for abdominal pelvic SFT is complete resection, the resection margin must be at least 1 cm away from the tumor to avoid tumor residue. Study from Demicco EG et al shown that geriatric patients with tumor size >15 cm have higher risk of metastasis,^[21]^ about 15% to 20% of SFT are malignant,^[15]^ particularly tumors above 10 cm, so for giant abdominal-pelvic SFT (>20 cm), complete resection has been shown to decrease the rate local recurrence and distant metastasis, up to 8%.^[22]^ However, it is up to 63% of malignant SFT developed local recurrence, repeated resection should be considered in cases of local recurrence.^[21]^ Study from Spitz FR et al shown that the 5-year survival rate of patients who underwent curative resection were higher (partial excision [79%]).^[23]^ Study demonstrated that the 5-year survival rate of patients who underwent curative resection ranging from 59% to 100%, and 10-year survival ranging from 40% to 89% have been observed when choosing complete curative resection.^[2,24–26]^ Therefore, curative resection could significantly improve the prognosis of giant abdominal-pelvic SFT and the prognosis of SFTs is often associated with resectability. Most of abdominal-pelvic SFT have a good prognosis without postoperative recurrence or metastasis, the recurrence of tumor was statistically related to capsule integrity for giant SFT. However, although the majority of SFT are benign, they exhibit the potential for malignant transformation. Thus, patients require long-term follow-up, considering the relatively good prognosis of SFT, adjuvant chemoradiation therapy after surgery is not recommended.^[2]^ Chemoradiation therapy is utilized in cases of non-resectable tumors.^[27]^ Increasing number of studies suggest that radiation therapy may reduce local recurrence, but no increase overall survival time.^[28,29]^ Based on the current study, combination therapy with bevacizumab and temozolomide was shown to be effective in the treatment of locally advanced SFT, temozolomide and dacarbazine can be active as single agents in SFT.^[30,31]^ Additional clinical studies are needed to better characterize the molecular pathways involved with SFT in order to treat it more effectively.

## 4. Limitations

The follow-up period was shorter in our study (6 months). Given the rarity of the disease, it is necessary to conduct a larger prospective study. Our conclusions are limited by the retrospective, observational nature of this study. Additionally, a microscopic pathological evaluation has its limitations in predicting the clinical behavior of this disease.

## 5. Summary

In conclusion, the diagnosis of giant abdominal-pelvic SFT relies on clinical symptoms, imaging features, and pathological examinations. The confirmation of the diagnosis is typically achieved through immunohistochemical testing, using CD34 and STAT-6 as the most specific and sensitive immunomarkers. Most of abdominal-pelvic SFTs are generally biologically benign, tumors above 20 cm are at a high risk of developing malignant, so complete surgical resection is the primary treatment option. However, giant abdominal-pelvic SFT may exhibit local recurrence or malignant transformation, emphasizing the importance of long-term follow-up after surgery.

## Acknowledgments

We would like to thank all the staff and nurses for their kind cooperation. We would also like to thank the patient.

## Author contributions

**Visualization:** Cao Li, Jianyang Yang, Hongli Chen, Lie Yang.

**Writing – original draft:** Cao Li, Jianyang Yang, Hongli Chen.

**Writing – review & editing:** Cao Li, Jianyang Yang, Hongli Chen.
